# Crosstalk between alcohol use disorder and obesity: two sides of the same coin?

**DOI:** 10.1038/s41380-025-03259-8

**Published:** 2025-09-27

**Authors:** Lorenzo Leggio, Mehdi Farokhnia, Paul J. Kenny, Marta Yanina Pepino, W. Kyle Simmons

**Affiliations:** 1 Clinical Psychoneuroendocrinology and Neuropsychopharmacology Section, Translational Addiction Medicine Branch, National Institute on Drug Abuse Intramural Research Program and National Institute on Alcohol Abuse and Alcoholism Division of Intramural Clinical and Biological Research, National Institutes of Health, Baltimore, MD USA; 2 Nash Family Department of Neuroscience, Icahn School of Medicine at Mount Sinai, New York, NY USA; 3 Department of Food Science and Human Nutrition, and Division of Nutritional Sciences, University of Illinois Urbana-Champaign, Urbana-Champaign, IL USA; 4 Department of Pharmacology & Physiology and OSU Biomedical Imaging Center, Oklahoma State University – Center for Health Sciences, Tulsa, OK USA

**Keywords:** Addiction, Neuroscience

## Abstract

Investigating similarities and differences between alcohol use disorder (AUD) and obesity is important because both AUD and obesity are public health concerns and share neurobiological and periphery-brain mechanisms. Furthermore, AUD and obesity often present with similar medical consequences related to organ damage, including liver and cardiovascular diseases. There is also growing evidence of changes in alcohol drinking in people who undergo bariatric surgery for obesity. In this non-systematic critical review, we identified relevant articles through PubMed searches, previous knowledge, and recursive reference searching. A librarian also used PubMed and Google Scholar for additional relevant articles, using terms such as alcohol, metabolic disorders, obesity, glucagon-like peptide-1 (GLP-1), bariatric surgery, and gut-brain axis. We provide an overview of the neurobiological, pathophysiological, neuroimaging, and clinical features related to the overlap and crosstalk between AUD and obesity. We also provide a summary of the currently approved medications for obesity and those for AUD and note the potential for some of these medications to work for both disorders. Specific to the latter point, we place emphasis on GLP-1 therapies, given their recent approval for weight loss and the growing evidence suggesting their potential efficacy for AUD and other addictions. We further review studies of the relationship between bariatric surgery and AUD and discuss potential mechanisms and future directions. In summary, studying the overlap between obesity and AUD may shed light on the mechanisms underlying the development and maintenance of both diseases. This knowledge, in turn, may help identify new therapeutic targets for AUD, and possibly comorbid obesity and/or other metabolic disorders.

## Introduction

Approximately 42% of adults in the United States have obesity [1], and 10% meet criteria for alcohol use disorder (AUD) [2]. An estimated 8 million adults (approximately 3% of the United States adult population) have obesity and drink alcohol heavily [3]. This comorbidity is particularly alarming given that AUD and obesity independently contribute to a host of comorbidities, including cardiovascular disease, diabetes, liver disease, and cancer [4, 5]. Although on the surface, both diseases seem to arise from ‘over-consumption’ – either of food or alcohol – their etiologies are far more complex than simply eating or drinking too much. Obesity, defined as a body mass index (BMI) greater than 30 kg/m^2^, results from the storage of excess energy in the form of adipose tissue [6]. How this excess energy comes to be stored by an individual, however, is not a simple matter, reflecting a multitude of biological, psychological, and environmental factors that predispose to or protect from obesity. AUD, in contrast, results from “a problematic pattern of alcohol use leading to clinically significant impairment or distress”, as defined by the DSM-5 [7]. As with obesity, how a pattern of alcohol use becomes problematic also reflects a complex interaction of biological, psychological, and environmental factors, which predispose to or protect from AUD.

Critically, research has shown remarkable overlap in the biological, psychological, and environmental determinants of AUD and obesity. AUD and obesity not only share overlapping neurocircuitry within the brain’s reward and appetitive pathways, but are also influenced by similar neuroendocrine and neuroimmune signaling pathways. As a result, AUD and obesity often respond similarly to treatments that target their common pathophysiologies, and interventions for one disease can often have consequences for the other. The goal of this narrative critical review is thus to investigate the similarities and differences in AUD and obesity’s causes and sequelae, with an eye toward identifying novel interventions that reduce the consequences of these two diseases for personal and public health.

We identified relevant articles through PubMed searches, previous knowledge, and recursive reference searching. A librarian also used PubMed and Google Scholar for additional relevant articles, using terms such as alcohol, metabolic disorders, obesity, glucagon-like peptide-1 (GLP-1), bariatric surgery, and gut-brain axis. Our search did not include specific inclusion/exclusion criteria, nor did we apply timeframe constraints. While this approach might not be free from potential bias, we envisioned this narrative overview of the literature as a *Critical Review*, that identifies dominant, overlapping themes of AUD and obesity.

## Environmental and genetic contributors to AUD and obesity

Owing largely to advancements in neuroscience and the development of effective biologically-based treatments (particularly pharmacotherapies), recent decades have seen remarkable changes in public opinion regarding the etiology of AUD and obesity [8–10]. Although significant stigma remains around both diseases [11–13], there has been a steady shift away from attributing AUD and obesity exclusively to individual failings of willpower or gluttony toward an understanding that both diseases arise from the multifactorial influence of genetics, environment, neurobiology, and culture.

The past two decades have witnessed an explosion of research on the genetics of obesity and AUD, with both shown to have substantial genetic heritability. The heritability of obesity is approximately 40–70%, with numerous genetic loci identified by genome-wide association studies [14]. Likewise, AUD is heritable (approximately 50%), although with a smaller number of loci as-yet identified [15, 16]. Although some epidemiological studies have provided support for a link between family history of alcohol problems and obesity, especially in women [17], the genetic correlation of AUD and obesity has been reported to be near zero [18, 19]. This is surprising given their overlapping pathophysiologies. More recently, evidence has emerged that the two diseases may have substantial genetic overlap, but with many of their shared gene variants having discordant (opposite) effects. For example, while the polygenic overlap of AUD and BMI is approximately 81%, only 48% of their shared loci have concordant effect directions [20, 21]. This finding may be critical to future efforts at identifying which obesity pharmacotherapies may be repurposed for AUD and vice versa.

As with genetics, significant progress has been made toward understanding how environmental and sociocultural factors influence AUD and obesity, although attention to specific environmental factors often differs between the two diseases. For example, the past decade has seen significant changes in the awareness that ‘built environment’ and access to nutritious foods (e.g., ‘food deserts’) greatly influence the development of obesity [22–24], but relatively less attention has been paid to evidence that access and availability of alcohol promotes problematic drinking [25, 26]. Furthermore, it is well-established that social factors and social networks play a role in both AUD and obesity; in fact, they influence alcohol consumption [27], and have effects that may be either obesogenic or protective [28–30]. More broadly, it is important to recognize that in addition to genetic and biological factors, social determinants of health, including socioeconomic status, employment, education, housing, and trauma history play a critical role in the development and maintenance of AUD and obesity. Therefore, prevention of chronic diseases like AUD and obesity should include the development of more and better community and national programs and policies aimed at addressing the social determinants of health that affect AUD and obesity and the associated medical consequences. This requires a comprehensive approach that includes not only biological but also social, environmental, and psychological factors [31, 32].

Notwithstanding their uneven application across obesity and AUD, we are encouraged by increased awareness that sociocultural factors considerably influence the development and course of both diseases. The present review, however, does not cover environmental, sociocultural, or genetic factors contributing to AUD and obesity, nor does it discuss related clinical phenotypes, including eating disorders, or the debate about whether obesity results from ‘food addiction’ [33, 34]. Rather, our focus is on how AUD and obesity’s shared neurobiological mechanisms, clinical features, and responses to extant treatments may inform our understanding of these two diseases, in the hope of highlighting new research opportunities and therapeutic targets that may ultimately lessen the burden of AUD and obesity.

### Clinical consequences of AUD and obesity

Both AUD and obesity lead to several medical consequences. AUD is not solely a mental health but a “whole body” disorder, playing a causal role in more than 200 diseases, injuries, and other health conditions [35, 36]. Similarly, obesity plays a causal role in the development of several diseases [37]. One might postulate a simple model whereby the shared negative health outcomes of AUD and obesity result from an increase in caloric intake with heavy alcohol consumption which then promotes the development of obesity. This is unlikely, however, as experimental and epidemiological findings suggest that excess energy from alcohol has a relatively greater effect on non-daily alcohol drinkers than in daily heavy drinkers [38].

AUD and obesity are responsible for significant organ damage and consequent medical complications, including (but not limited to) on the liver, gut, pancreas, nervous system, cardiovascular system, endocrine system, and immune function, plus increased risk for other medical conditions like cancers [36, 39]. The mechanisms underlying these interactions are complex and not fully understood; some are distinct to AUD or obesity, while others overlap. Some alcohol-associated organ damage is related to the direct toxic effects of alcohol on cells, tissues, and organs, while some obesity-related medical consequences are attributed to increased fat mass and/or increased release of peptides from enlarged fat cells [37]. By contrast, inflammation, and especially low-grade inflammation, as well as dysregulated immunity (e.g., altered leucocyte counts as well as cell-mediated immune responses), is shared between AUD and obesity, contributing at multiple levels to their related medical complications, but also representing potential treatment targets [40, 41]. For comprehensive reviews on mechanisms and signaling pathways related to these complications, see e.g. [37, 39] for obesity and [36] for AUD. Some of the medical consequences linked to AUD or obesity arise from their comorbidities, either directly or indirectly. For example, both AUD and obesity are directly responsible for hypertension and cardiomyopathies, with hypertension potentially further exacerbating cardiomyopathies.

Clinically, there are some distinct features in the management of patients with AUD and those with obesity. Obesity-related medical consequences are typically chronic in nature and acute manifestations are secondary to the underlying organ damage. For example, obesity leads to cardiovascular disease (CVD), which in turn may lead to acute myocardial infarction (MI). Another example is that obesity leads to diabetes which in turn may lead to e.g., acute diabetic ketoacidosis. While these scenarios are also observed in people with AUD (AUD → CVD → MI; AUD → diabetes → acute diabetic ketoacidosis, etc.), AUD and alcohol drinking are also directly responsible for acute medical conditions, including after acute alcohol intoxication [42]. For example, while AUD chronically increases the risk of diabetes, acute alcohol intoxication (even in absence of AUD) may cause hypoglycemia. In addition to alcohol-related gastrointestinal chronic conditions like peptic ulcer disease and esophagitis, acute alcohol intoxication may lead to acute esophagitis and gastritis with severe nausea and vomiting and in some cases life-threating complications due to e.g., bleeding and Mallory-Weiss syndrome (tear or laceration of the gastroesophageal junction) [42]. These medical emergencies are often the result of an ‘acute on chronic’ worsening of alcohol-associated medical complications, e.g., acute on chronic gastritis, hepatitis, pancreatitis.

Given that AUD and obesity are common and frequently co-exist, the related medical consequences often arise from their additive or synergistic effects. For example, hypertension and cardiomyopathy are common in people with obesity and/or AUD; weight loss as well alcohol abstinence or even reduction are associated with improvement in cardiovascular comorbidities [37, 43, 44]. Importantly, patients with both AUD and obesity are even more likely to have comorbid cardiovascular complications due to the dual etiology.

Hepatic diseases offer another cogent example of the dual etiology of AUD and obesity in organ-related damage and consequences. People with alcohol-associated cirrhosis have a much lower life expectancy than the general population (on average 14 years shorter for men and 16 years shorter for women) [45]. Not only is alcohol a main cause of chronic liver disease and acute liver failure, but also with the development of new effective treatments for hepatitis C, alcohol has now become the leading cause of cirrhosis and the leading indication for liver transplantation in the United States [46]. Like alcohol use, obesity in the United States has also increased, leading to an increase in the previously named non-alcoholic fatty liver disease (NAFLD) [39, 47]. Several studies have shown accelerated liver disease in people with metabolic syndrome (central adiposity, diabetes, hypertension, and dyslipidemia) who also drink alcohol [48]. For example, a study from Northern Europe showed a ~ 7‐fold increase in incidence of severe liver disease in people with greater central adiposity who also consumed >1 and >2 alcohol-containing drinks/day for women and men, respectively [49]; such increase approached 20-fold in people with diabetes versus those without. In other words, not only are AUD and obesity two leading causes of liver diseases, but also their frequent comorbidity contributes to accelerated development and worsening of liver disease. The increased knowledge of overlapping biological processes that contribute to both NAFLD and alcohol-associated/related liver disease (ALD), coupled with the increased clinical and public relevance of this dual etiology, has led to a recent effort across multiple international liver societies to develop a new nomenclature. NAFLD has been replaced with metabolic dysfunction-associated steatotic liver disease (MASLD), and a new category, outside of pure MASLD, was created and named MetALD to describe those with MASLD who consume excessive amounts of alcohol (140 and 210 g/week for women and men, respectively) [50]. This new nomenclature acknowledges the common clinical feature of concomitant metabolic- and alcohol-related contributions to hepatic steatosis and subsequent liver inflammation, damage, and degeneration. This interdisciplinary approach may serve as a model for studying other medical conditions due to AUD and obesity comorbidity.

It is also key to consider the important role of sex differences in the development and clinical manifestations of both obesity and AUD, including differences in alcohol and food metabolism. In fact, not only sex differences exist in obesity and AUD per se, but also treatment response may vary as a function of sex. Furthermore, growing evidence shows sex differences in the medical consequences of these chronic diseases, including obesity-related conditions like obstructive sleep apnea and heart failure [51], and AUD-related conditions like ALD [46].

## Neuroscientific knowledge and shared mechanisms of AUD and obesity

Both AUD and obesity are characterized by compulsive consummatory behaviors that persist despite adverse consequences, at least partially consistent with intersecting neurobiological mechanisms [33, 52]. Emerging research highlights common brain circuitries that contribute to these behaviors [33, 52]. Understanding the circuit-based mechanisms of AUD and obesity can provide valuable insights into their shared pathophysiology. This section will put special emphasis on GLP-1-related mechanisms in obesity and AUD, given the growing evidence of its important role in both disorders and the related treatment implications, as discussed later.

### Mechanisms related to reward processing and dopamine-related pathways

Consumption of alcohol and energy-dense palatable foods increases the activity of dopamine neurons in ventral midbrain, leading to enhanced dopamine transmission in the nucleus accumbens (NAc) and other areas of the striatum. Dopamine-dependent and -independent signaling in the NAc and dorsal striatum regulate alcohol and palatable food consumption. Furthermore, prolonged alcohol drinking and weight gain resulting from overeating of energy-dense food are associated with deficits in mesostriatal dopamine transmission [53, 54], possibly involving mechanisms that originate in the gastrointestinal tract [55–58]. Positron emission tomography (PET) studies have established that striatal Dopamine D2 receptor (D2R) availability is decreased in individuals with AUD and obesity [59–64]. Disrupted D2R signaling in the striatum is thought to play an important role in AUD and diet-induced obesity. Indeed, the TaqIA allele of the *DRD2/ANKK1* gene locus, which results in reduced striatal D2R expression, is associated with increased predisposition to AUD and obesity [65–67]. D2R gene knockout mice show increased aversion to alcohol, reduced alcohol consumption, and reduced sensitivity to the locomotor impairing effects of alcohol [68]. Furthermore, conditional deletion of D2Rs from striatal medium spiny neurons in mice reduces their sensitivity to the locomotor-impairing effects of alcohol and precipitates compulsive-like alcohol consumption, as reflected by drinking despite negative consequences [69]. In rats, RNA interference-mediated knockdown of D2Rs in the striatum similarly rendered consumption of palatable food similarly resistant to the appetite-suppressing effects of punished-associated conditioned stimuli [70]. These findings suggest that striatal D2R signaling contributes to the compulsive nature of both AUD and obesity. Notably, GLP-1 receptor agonists (GLP-1RAs) modulate dopamine signaling in the striatum and other brain regions [71–83], which is thought to contribute to their inhibitory effects on alcohol and food consumption.

### Mechanisms related to stress, including hypothalamic and extrahypothalamic pathways

Stress plays an important role in the etiology and maintenance of both AUD and obesity [84–86]. The hypothalamic-pituitary-adrenal (HPA) axis mediates physiological and behavioral adaptations to prolonged stress, and chronic activation of this system can drive maladaptive behaviors, including over-consumption of alcohol and palatable food [87, 88]. In AUD, stress-induced activation of the HPA axis is thought to enhance alcohol craving and consumption in part to obtain the transient stress-alleviating actions of alcohol [89, 90]. Similarly, chronic stress is associated with overeating, particularly of palatable high-energy foods, a phenomenon sometimes referred to as “comfort eating” [91–93]. The central nucleus of the amygdala (CeA) is a core component of the extended amygdala and a critical node linking stress and reward pathways [94]. Dysregulated neural activity in the CeA is thought to contribute to stress-enhanced consumption of alcohol and palatable food through HPA-dependent and -independent mechanisms [95–99]. The bed nucleus of the stria terminalis (BNST) is another component of the extended amygdala heavily implicated in stress-related alcohol and palatable food consumption [100–105]. Neurons in the CeA and BNST express GLP-1Rs, and both structures are thought to regulate the effects of GLP-1RAs on the HPA axis and consummatory behaviors [106–114].

The hypothalamus is essential for maintaining energy homeostasis [115]. Major hypothalamic nuclei, including the arcuate nucleus, paraventricular nucleus, and lateral hypothalamus, integrate energy-related signals from peripheral hormones, including leptin, ghrelin, insulin, glucose-dependent insulinotropic polypeptide (GIP), and glucagon, to regulate feeding behavior and energy expenditure [115]. Weight gain resulting from the overconsumption of palatable food is associated with structural and functional adaptions in the hypothalamus, particularly in the lateral hypothalamus, which contribute to the development of compulsive-like food intake [116–120]. Alcohol drinking also affects hypothalamic function and can alter hunger and satiety signals to promote food consumption [121–123], while hunger-related hormones that act on the hypothalamus can stimulate alcohol craving and intake [122, 124]. The lateral hypothalamus also regulates alcohol seeking and consumption [125–128]. GLP-1R signaling in the lateral hypothalamus and other hypothalamic nuclei regulates alcohol and food consumption [129–132].

### Mechanisms related to satiety and appetite

The nucleus of the solitary tract (NTS) and area postrema (AP) in the hindbrain contain transcriptionally heterogeneous populations of neurons that regulate satiety-related suppression of appetite, food avoidance, malaise, and nausea [133–136]. Diet-induced adaptations in NTS and AP neurons are thought to contribute to the development of overeating and obesity [133, 137–139]. Lesioning AP neurons increases alcohol consumption in rats through an unclear mechanism independent of alcohol-induced aversion or nausea [140]. Little is currently known about the role of NTS neurons in regulating alcohol consumption, although these cells regulate the consumption of other addictive drugs [141]. Notably, the NTS contains a population of preproglucagon-producing (PPG+) neurons that are the predominant or exclusive source of GLP-1 in the brain [142–145]. The NTS and AP also contain high concentrations of GLP-1Rs [146]. It was recently established that activation of GLP-1R-expressing (GLP1R+) neurons in the NTS triggered satiety and reduced food intake independent of aversion, while activation of GLP1R+ neurons in the AP elicited aversion accompanied by suppressed food intake. Little is currently known about the role of GLP1R+ neurons in the NTS and AP in regulating compulsive alcohol and food consumption.

In addition to PPG+ neurons in the NTS, GLP-1 is also produced by entero-endocrine cells in the gastrointestinal tract [147–149]. Peripheral GLP-1 is rapidly degraded by dipeptidylpeptidase-4 (DDP-4) and is unlikely to accumulate to levels sufficient to stimulate brain GLP-1R signaling [142–145]. However, peripheral GLP-1 can stimulate GLP-1R-expressing nodose (vagal) sensory neurons that innervate the gastrointestinal tract and hepatic portal vein and transmit feeding-related information to the NTS [150–153]. GLP1R+ nodose neurons play an important role in regulating meal termination and the appetite-suppressing effects of peripheral GLP-1 [154–159]. Impaired transmission of satiety-related information from the gastrointestinal tract to the NTS by nodose neurons may contribute to diet-induced obesity [160–162]. Nodose sensory neurons have also been implicated in the regulation of alcohol consumption [163], although few studies have directly investigated peripheral sensory mechanisms of alcohol reinforcement. Currently, it is unclear whether the suppression of alcohol and palatable food consumption by GLP-1RAs results from activation of GLP1R+ nodose neurons, stimulation of GLP1R+ neurons in the brain or a combination of both.

### Prefrontal cortex mechanisms related to cognitive control/behavioral inhibition

In individuals with obesity and substance use disorders including AUD, increased prefrontal cortex (PFC) activity has been noted in responses to both food and drug cues respectively [164, 165]. Given this and the established role of the PFC in cognitive control and attention [166], it is notable that some studies have found evidence that both conditions are associated with decreased behavioral inhibition in the presence of either food- or alcohol-stimuli as well as altered PFC activity (particularly in the dorsolateral PFC and anterior cingulate cortex) while performing food- or alcohol-related cognitive control neuroimaging tasks (for review, see [167]). Regardless of whether this PFC dysfunction is a cause or consequence of either obesity or AUD, its implications for treatment adherence and persistence are clear: whether in weight loss for obesity or reducing alcohol consumption in the context of AUD, more cognitive control and behavioral inhibition is better than less. Cognitive and behavioral treatments that emphasize the development of cognitive control and behavioral inhibition have demonstrated efficacy in promoting weight loss and reducing alcohol consumption [168–170], and neuromodulation of the dorsolateral PFC via transcranial magnetic stimulation (TMS) has exhibited potential efficacy for both conditions as well [171, 172] (see neuromodulation section below).

### Neuroimaging studies of AUD and obesity

There exist expansive human neuroimaging literature examining obesity and AUD. Although a thorough analysis of the similarities and differences among these literatures is beyond the scope of this review, a few general comments are worth making here. First, PET and functional magnetic resonance imaging (fMRI) studies using visual, olfactory, and gustatory stimuli overwhelmingly find that perception of food and alcohol cues activates overlapping distributed neural networks. These include striate and extrastriate visual areas to process visual features, gustatory and interoceptive regions in the insula to predict taste and the effects of food and alcohol on the body, and appetitive and reward-related regions in striatum and orbitofrontal cortex that represent the motivational salience and anticipated hedonic value of food and alcohol cues [164, 173–177].

Although neuroimaging studies have largely examined obesity and AUD phenotypes separately, their findings are generally overlapping and, within the limitations of neuroimaging’s spatial and temporal resolution, highly consistent with the preclinical studies reviewed above. For example, human PET and fMRI studies do not typically report engagement of sub-voxel-sized hypothalamic or brainstem sub-nuclei that powerfully impact food and alcohol consumption, but they do point to larger structures highlighted in preclinical studies. These include the ventral and dorsal striatum, anterior cingulate, orbitofrontal cortex, and amygdala, which typically exhibit greater activation to food or alcohol stimuli in people with obesity or AUD compared to controls [164, 173, 175, 178]. This presents an important question: if human neuroimaging is only able to measure a subset of the known circuitry underlying appetitive responses to food and alcohol, why bother? The answer is that it is challenging to reliably measure certain important phenomena in animals, such as the phenomenological experience of food or drug craving during voluntary abstinence [179–181]. Here, again, evidence suggests that the neural substrates of alcohol and food craving are largely overlapping, encompassing the ventromedial/orbitofrontal PFC, dorsal and subgenual anterior cingulate cortex, ventral striatum, parietal and temporal areas, cerebellum, and amygdala, with machine-learning classifier algorithms trained on one class of stimuli (e.g., food) reliably predicting craving for the other class (e.g., alcohol), and vice versa [182].

## Treatments of AUD and obesity

Behavioral and pharmacological interventions, in addition to lifestyle modifications, constitute the mainstream treatment options to manage AUD as well as obesity.

For AUD, Alcoholics Anonymous and 12-Step facilitation treatments and other similar programs have historically played a key role in helping people maintain alcohol abstinence and reduce alcohol drinking [183]. Evidence-based behavioral treatments for AUD encompass brief interventions (including as part of the Screening, Brief Intervention, and Referral to Treatment (SBIRT) approach), motivational interviewing, cognitive behavioral therapies (CBT), contingency management, and mindfulness-based interventions [184–186]. Medications approved by the Food and Drug Administration (FDA) to treat AUD are naltrexone, acamprosate, and disulfiram. A few other medications have also shown efficacy and are sometimes used off-label for AUD, including topiramate, gabapentin, baclofen, and varenicline [186–188]. Optimal approach, albeit rarely implemented in clinical practice, would include pharmacotherapy plus an evidence-based behavioral therapy [189].

Many patients with AUD or other substance use disorders report an increase in food cravings and weight gain upon cessation of alcohol consumption and drug use [190–192]. The various accounts offered for this phenomenon generally fall into two camps [191]. One hypothesis asserts that individuals with substance use disorders have a strong neurobiological propensity for compulsive/addictive behaviors that include overeating, and that food motivation, which is often suppressed by the effects of alcohol and drugs, returns in force upon abstinence. An alternative ‘addiction transfer’ hypothesis asserts that following cessation of substance use, individuals may replace drug rewards with other substances (e.g., food) which stimulate similar neurobiological reward pathways. Although a full discussion of this topic is beyond the scope of this review, it is noteworthy that both accounts ground the phenomenon of increased food craving following cessation of alcohol or drug use in the shared neurobiological bases of obesity and alcohol and other substance use disorders.

The main treatment modalities for obesity considerably overlap with those used for AUD. Behavioral treatments for obesity are often delivered as part of multicomponent programs such as Weight Watchers and Diabetes Prevention Program and include goal setting, monitoring and adjustment of calorie intake and physical activity, stimulus control, stress management, and CBT [193–195]. FDA-approved medications for obesity include phentermine, phendimetrazine, and diethylpropion for short-term use and phentermine-topiramate, naltrexone-bupropion, orlistat, and more recently the GLP-1RAs liraglutide and semaglutide, and the GLP-1/GIP dual receptor agonist tirzepatide for long-term use. Other medications such as metformin, topiramate, and bupropion are also used off-label for weight loss [195, 196]. It is particularly intriguing that pharmacotherapies for AUD, e.g., naltrexone and topiramate, also reduce appetite and promote weight loss, a clinical observation consistent with the neurobiological overlap between AUD and obesity discussed before. Moreover, emerging research suggests that feeding-related and metabolic endocrine pathways such as ghrelin and GLP-1 could serve as promising pharmacotherapeutic targets for AUD [197].

The development, approval, and rapid clinical adoption of GLP-1RAs like semaglutide and tirzepatide has revolutionized the medical management of obesity and comorbid conditions such as diabetes, obstructive sleep apnea, liver and cardiovascular diseases [198, 199]. Following food intake, GLP-1 stimulates insulin secretion and inhibits glucagon release from the pancreas, hence regulating glucose homeostasis. Activation of GLP-1Rs also delays gastric emptying, reduces gastrointestinal motility, and overall suppresses appetite and food intake [200]. Compared to the first generation of GLP-1RAs (e.g., exenatide), newer GLP-1RAs (e.g., semaglutide) and poly agonists (e.g., tirzepatide, retatrutide) have higher affinity for the GLP-1R, longer half-lives, and are more potent, leading to high efficacy for glucose control and weight reduction [201–203].

Growing evidence suggests that GLP-1RAs also reduce alcohol use and may be repurposed to treat AUD. Preclinical experiments across different species, using various models and conducted by independent laboratories, show that central or peripheral administration of GLP-1RAs reduce alcohol intake and other related outcomes such as alcohol-induced conditioned place preference [197, 204, 205]. While initial studies were done with first-generation GLP-1RAs, more recent work has consistently found a robust effect with semaglutide reducing binge-like alcohol drinking in mice [110], operant self-administration, alcohol intake, and relapse-like drinking in rats [72, 110], and alcohol consumption in alcohol-preferring vervet monkeys [206]. In addition to their impact on appetite and consummatory behaviors [207, 208], several mechanisms have been proposed to mediate GLP-1RAs’ effects on alcohol intake, including interactions with reward-related pathways [209–212], stress [213–215], cognition and neuroprotection [216–218], pain and aversion [113, 219, 220], and inflammation [221–223].

Clinical work on the potential efficacy of GLP-1RAs for AUD is under way. In a 26-week clinical trial, exenatide 2 mg/week had no significant effect on the primary outcome of alcohol drinking in the full sample (dropout rate: 54.3%), while it reduced alcohol cue-reactivity (fMRI) and dopamine transporter availability (SPECT) in the brain. Exploratory analyses found a significant reduction in alcohol intake among exenatide-treated participants who had a BMI > 30 kg/m^2^, whereas an opposite effect was found among those with a BMI < 25 kg/m^2^ [224]. In a secondary analysis of a 12-week smoking cessation trial, dulaglutide 1.5 mg/week significantly reduced weekly alcohol consumption (most participants had a BMI > 29.9 kg/m^2^, and all received varenicline and behavioral counseling) [225]. Furthermore, recent case series and analyses of social media posts indicate that patients receiving semaglutide or tirzepatide report beneficial effects on alcohol-related outcomes [226–229]. With the exponential growth in clinical use of GLP-1RAs, pharmacoepidemiological studies have also analyzed the association between receipt of GLP-1RAs and alcohol-related outcome, using real-world electronic health records. A recent study using aggregate data from 61 healthcare organizations found that receipt of semaglutide, compared to other non-GLP-1RA anti-obesity and anti-diabetes medications, was associated with reduced risks of incident and recurrent AUD [230]. Other large-scale studies using different datasets have also found beneficial effects with GLP-1RAs on other outcomes, including the frequency and quantity of alcohol use as measured by alcohol use disorders identification test-consumption (AUDIT-C) [231], alcohol-related events [232], intoxication [233], and hospitalizations [234]. While these observational findings are promising, randomized controlled trials (RCTs) are needed to draw conclusions [235, 236]. A recent RCT showed that low-dose semaglutide was effective, compared to placebo, in reducing laboratory alcohol self-administration, as well as drinks per drinking days, heavy drinking, and alcohol craving during the 2-month trial duration [237]. Additional ongoing clinical trials are studying GLP-1RAs, mostly semaglutide, in individuals with AUD and comorbid conditions. Beyond the GLP-1 system, several other feeding/metabolic endocrine pathways have also shown promise as potential pharmacotherapeutic targets for AUD and/or obesity, including (but not limited to) ghrelin [238–243], orexins [244–248], amylin [249–252], and fibroblast growth factor 21 [253–256]. Further research is needed to investigate the safety and efficacy of novel medications targeting these and other systems for the treatment of AUD and/or obesity.

Beyond behavioral and pharmacological treatments, growing evidence supports the potential use of neuromodulation techniques such as repeated TMS (rTMS), transcranial direct current stimulation (tDCS), deep brain stimulation, and vagus nerve stimulation, as emerging treatments for addictions, including AUD. Among these promising neuromodulation approaches, rTMS is the most investigated technique, using several coils (including the most common 8-coil and the H-coil for deep-TMS) and targeting different brain regions, the most common being the dorsolateral PFC. Of note, the salience network represents a hub of several circuits and networks associated with AUD and recent evidence suggests that rTMS targeting the salience network may be particularly promising for AUD [257]. Similarly, growing evidence suggests a role of neuromodulation approaches for obesity. While previous systematic reviews did not observe clear evidence for these approaches with obesity [258], more recent meta-analytical evidence based on RCTs supports that rTMS as well as tDCS lead to weight loss in people with obesity, possibly via a reduction in food craving [172]. Nonetheless, larger RCTs are needed to assess the potential of rTMS and other neuromodulation approaches for treating AUD and obesity.

## Bariatric surgery for obesity and increased risk of AUD

Bariatric surgery is the most effective long-term treatment for severe obesity [259], with over two million procedures in the past decade in the United States [260]. As prevalence of severe obesity grows and new guidelines endorse these surgeries for pediatric patients [261], procedure volumes are expected to climb. In the United States today, sleeve gastrectomy (SG) accounts for ~70% and Roux-en-Y gastric bypass (RYGB) for 27% of cases [262]. Laparoscopic gastric banding (LAGB), has dwindled to less than 1% of surgeries because of modest weight loss and greater long-term weight regain [262]. LAGB merely constricts gastric volume, whereas SG (resection of ~70–80% of the stomach, leaving a tubular remnant) and RYGB (creation of a small gastric pouch anastomosed to the jejunum) fundamentally change gastrointestinal anatomy and physiology. These alterations enhance signals such as GLP-1 [263, 264] reshaping appetite, reward processing, and glycemic control, earning SG and RYGB the label “metabolic surgeries.”

The concept of surgically treating obesity traces back to mid-20th-century observations that patients who underwent partial/total gastrectomy for ulcers or cancer often shed substantial weight [265]. Those same gastric resections, however, were accompanied by unexpectedly high rates of AUD [266, 267]. Only in the late 2000s was a similar association recognized for bariatric procedures [268].

Preclinical work supports a causal link. In several studies, rats with obesity that received RYGB consumed more alcohol or worked harder for oral [57, 269, 270] or intravenous [271, 272] alcohol than sham-operated controls suggesting a biological driver of postoperative alcohol misuse. In rodents, SG does not increase alcohol consumption [273–275] (for a detailed review, see also [276]). These divergent findings in rodent models hint that the magnitude and/or mechanisms related to AUD risk may vary by procedure.

One of the earliest clinical signals came from an alcohol treatment program that noted disproportionate enrollment of RYGB patients [277]. Two prospective cohorts in the United States subsequently documented a near-doubling in the incidence of AUD [278] and increased frequency of alcohol use [279] two years after RYGB versus LAGB. At five-year follow-up, ~20% of RYGB patients met AUD criteria determined by the Alcohol Use Disorders Identification Test [280]. A Swedish registry (n = 12,277, 1980–2006) showed that RYGB was associated with a two-fold greater risk of inpatient AUD treatment than LAGB [281] and the controlled Swedish Obese Subjects study confirmed an elevated risk after gastric bypass [282]. A Danish national cohort (n = 14309; 95% RYGB) extended these findings, revealing a 7% higher hazard of AUD five years post-surgery compared with both pre-surgery values and nonsurgical controls with obesity [283]. Because SG became widespread after 2014, the data set in humans is smaller than that for RYGB, but several [284–286] though not all [287–289] studies suggest SG approximates RYGB in raising AUD risk, particularly for de novo post-operative alcohol problems.

Multiple, potentially interacting mechanisms have been proposed for the increased risk of AUD after metabolic surgery. First, SG and RYGB accelerate alcohol absorption and reduce first-pass metabolism by quickening gastric emptying, which result in earlier and higher blood alcohol concentration (BAC) peaks [290–295]. Women can surpass the legal driving BAC limit (0.08%) within minutes of consuming less than two standard drinks [293, 294, 296]. Rapid central (brain) delivery is strongly linked to addictive potential [297]. Furthermore, a BAC of 0.08% meets the definition for binge alcohol drinking, which typically occurs after women consume 4 (and men 5) standard drinks within 2 h [298]. Therefore, RYGB/SG surgeries can turn moderate-seeming intake into pharmacological binge episodes that foster alcohol tolerance, which can further drive AUD [299, 300]. The magnitude of the alcohol-pharmacokinetic changes that follow metabolic surgeries is likely underestimated in studies that use breath analyzers, which cannot capture the very early post-ingestion BAC peaks after RYGB and SG [294, 301, 302].

Second, metabolic surgeries dramatically shift gut-peptide profiles that modulate reward circuits. GLP-1 rises sharply post-prandially [303] whereas fasting ghrelin falls (in SG and at least early in RYGB) [304, 305]. In rodent models, enhanced sensitivity of central ghrelin receptor (the growth hormone secretagogue receptor (GHSR)) mediates heightened motivation for alcohol [271]. Remarkably, 40-50% of patients with high-risk alcohol use before surgery reduced drinking within the first year after RYGB surgery [278, 306–308] yet AUD risk surges two or more years later [278, 279, 284]. This temporal pattern supports the idea of dynamic neuroadaptations —initial peptide changes may blunt reward [309], but chronic reductions in ghrelin, for example, could up-regulate GHSR and later amplify alcohol seeking [303].

Third, the marked post-operative caloric restriction inherent to SG and RYGB could sensitize mesolimbic dopamine pathways, paralleling findings that food deprivation enhances the rewarding effects of drugs in humans and animal models [310–312]. Finally, obesity itself is associated with reward-circuit alterations resembling those caused by AUD [64, 313], as reviewed above. Once highly palatable foods become less reinforcing after surgery, patients may “transfer” reward seeking to alcohol [314].

Key knowledge gaps remain. SG is linked to increased AUD in humans but not in rodent models; resolving whether species differences, surgical techniques, or alter alcohol pharmacokinetics accounts for this discrepancy is essential. Another critical research area involves surgery in the pediatric population, who as teenagers and young adults will have a higher baseline propensity for binge drinking. The Teen-LABS study found that 47% of adolescents who underwent metabolic surgery screened positive for excessive alcohol drinking or alcohol-related problems eight years later [315]. As metabolic surgery expands in youth, clinicians must recognize altered alcohol metabolism and implement age-appropriate screening and counseling. Finally, growing evidence links post-bariatric AUD to alcohol-related organ injury, including liver disease [316, 317]. Systematic screening algorithms as well as targeted preventive and therapeutic strategies are urgently needed to maximize the metabolic benefits of surgery while mitigating its unintended addiction-related harms.

## Open questions, challenges, and final remarks

**Fig. 1:**
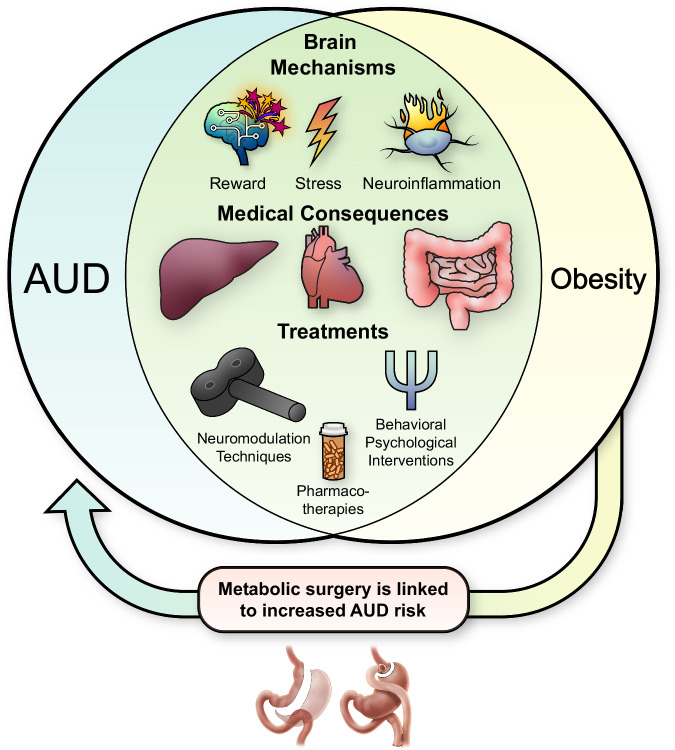
Overlaps, similarities and differences between alcohol use disorder and obesity. Development and course of alcohol use disorder (AUD) and obesity share several central and peripheral mechanisms, such as dysregulations in reward processing, stress pathways, consummatory behavior, inflammation, immunity, and endocrine systems. Some of the key brain regions with shared neurocircuitry between AUD and obesity include ventral and dorsal striatum, extended amygdala, prefrontal cortex, hypothalamus, nucleus of the solitary tract, and area postrema. Genetic, environmental, and psychosocial factors, some shared and some distinct, also contribute to the natural history of AUD and obesity. While some comorbidities are more prevalent or specific to one condition, AUD and obesity lead to several overlapping medical consequences, such as cancers, liver, gastrointestinal, and cardiovascular diseases, and disruptions in endocrine and immune systems. In addition to lifestyle modifications, treatment options for AUD and obesity include a range of interventions. Some behavioral psychological interventions such as motivational interviewing and cognitive behavioral therapies are used for both conditions, while others are more specific to one (e.g., contingency management for AUD). Food and Drug Administration (FDA)-approved and off-label medications used to treat AUD and obesity also have some commonalities (e.g., naltrexone, topiramate) and growing evidence suggests that, pending additional evidence, anti-obesity GLP-1 therapies (e.g., semaglutide, tirzepatide) have the potential to be repurposed for AUD. Furthermore, growing evidence supports the potential use of neuromodulation techniques such as repeated transcranial magnetic stimulation (rTMS) and others, as emerging novel treatments for obesity as well as addictions, including AUD. Finally, preclinical and human studies suggest a link between metabolic surgeries and increased risk of AUD.

This narrative critical review provides an overview of the overlapping features of AUD and obesity (Fig. 1). AUD and obesity are two chronic endemic diseases which share common neurobiological mechanisms, identified in animal models and human neuroimaging studies, and both lead to many similar medical consequences. Another important evidence of the overlap between AUD and obesity is that some treatment modalities work for both weight loss and alcohol reduction. Unfortunately, both AUD and obesity are dramatically under-treated and stigmatized. Our healthcare structure is based on organ-specific specialized approaches and the focus is often treating medical consequences rather than root causes (e.g., treating hypertension but ignoring the underlying AUD and/or obesity). Second, many healthcare providers are not trained in the screening, diagnosis, and management of these diseases, although the addiction medicine / addiction psychiatry and obesity medicine fields are growing. Screening for alcohol problems is not routinely done in primary care. While weight is routinely measured, there is rarely a focused discussion about it. Third, patients often do not seek treatment for their obesity or AUD, due to a variety of reasons, including denial, lack of awareness that effective treatments exist, and the stigma surrounding obesity and AUD. People with obesity, as well as those with AUD, are subject to significant stigma with severe personal, societal, and healthcare consequences [318, 319]. Stigma causes physical and psychological harm to patients, and affected individuals are less likely to receive adequate evidence-based care and treatments [318, 319].

The development of GLP-1RAs as new effective treatments for weight loss has revolutionized medicine. There is much that can be learned and applied to the addiction field, including for AUD. A few examples follow.

1. The treatment of obesity is changing significantly because of the increased acceptance that it is medical condition, rather than simply a lifestyle choice. Likewise, it is critical to promote awareness of addiction, including AUD, as a chronic relapsing and treatable brain disease [320–322]. This notion does not negate the important role of environmental and social factors in AUD, but the medical disease model of addiction highlights the need to develop new treatments, including pharmacotherapies and neuromodulation approaches.
2. It is crucial to reduce stigma not only at the individual patient level but also at the organizational and structural levels, including among healthcare providers and policy makers.
3. In addition to increasing clinicians’ awareness of AUD as a medical disorder, an important lesson to learn from the “GLP-1” era is that these complex multifaceted medical conditions need to be studied and managed by multidisciplinary teams, not just a niche of highly specialized providers. The use of GLP-1RAs is impacting not only obesity medicine, but also primary care, cardiology, nephology, hepatology, and surgery. Similarly, AUD is a “whole body” disease, and the engagement of diverse clinicians/disciplines is critically important to improve the screening, diagnosis, and management of people with AUD. Initial efforts in this direction are under way between the addiction and hepatology fields in managing people with AUD and ALD [46–48, 323]; such approaches must be expanded across other relevant disciplines. Specific to the current efforts aimed at testing GLP-1RAs for addictions, including AUD, safety considerations are also of paramount importance. For example, patients with AUD may have malnutrition or metabolic conditions that may be worsened by these drugs e.g., loss of free fat mass. The latter is particularly important to keep in mind in patients with AUD and comorbid ALD who often present with sarcopenia. Furthermore, alcohol is one of the main causes of pancreatitis and GLP-1RAs are associated with increased risk of pancreatitis, therefore extra caution is needed in this patient population.
4. The often-prohibitive costs associated with the newer GLP-1RAs for obesity serve as a reminder that similar equity issues may rise for people with AUD, should these or other medications be proven to be effective in AUD [235]. Therefore, it will be imperative to ensure that future new effective treatments have a wide implementation among people with AUD.
5. Given some challenges with accessing GLP-1RAs, e.g., due to shortage in the market, prohibitive costs and other factors, the obesity medicine field has observed an uptake in the prescription of other approved medications for obesity. While there is a need for more pharmacotherapies for AUD, we already have FDA-approved medications for AUD (acamprosate, disulfiram, naltrexone). Despite meta-analytical evidence that naltrexone and acamprosate are effective in people with AUD [324], it’s estimated that <2% of people with AUD in the United States receive an FDA-approved medication for AUD [325]. Therefore, it’s critical to increase the use of FDA-approved medications for AUD for those seeking treatment, while the research field continues to work toward the discovery and development of additional effective treatments.
